# Distributional Patterns of Aquatic Empididae (Diptera) along an Elevational Diversity Gradient in a Low Mountain Range: An Example from Central Europe

**DOI:** 10.3390/insects12020165

**Published:** 2021-02-15

**Authors:** Iwona Słowińska, Radomir Jaskuła

**Affiliations:** Department of Invertebrate Zoology and Hydrobiology, Faculty of Biology and Environmental Protection, University of Lodz, Banacha 12/16, 90-237 Łódź, Poland; radomir.jaskula@biol.uni.lodz.pl

**Keywords:** Hemerodromiinae, Clinocerinae, Empididae, species richness, altitudinal distribution, elevational diversity gradient, habitat preferences, Pieniny Mts., Poland

## Abstract

**Simple Summary:**

The insect distribution and diversity depend on many different abiotic and biotic factors, which is especially well documented in the high mountains but has not been studied in detail in the low mountain massifs. We studied 17 different macro and microhabitat factors that influence the altitudinal distribution of 40 Hemerodromiinae and Clinocerinae species in the Pieniny Mts., Poland. This is the first such study in Central Europe and one of only a few in the world. The results clearly show that species richness and distribution of Hemerodromiinae and Clinocerinae species are changing with the elevational gradient, with a monotonic decline in species richness with increasing elevation observed for the first subfamily and the hump-shaped distribution pattern noted for the second subfamily, as well as the size of the stream/river and the surrounding area in species distribution in the Pieniny Mts.

**Abstract:**

The two subfamilies Hemerodromiinae and Clinocerinae, also known as aquatic dance flies, are a group of small predatory insects occurring mainly in mountainous areas and the northern temperate. However, very little is known about distribution patterns for most of the species. Habitat preferences for 40 aquatic empidid species were analysed in the Pieniny Mts., Poland. Forty-six sampling sites from a major part of this relatively low mountain massif (400–770 m) were chosen, for which 17 micro and macrohabitat environmental variables were measured including both abiotic (altitude, stream mean width and depth, and shading) and biotic factors (13 dominant plant communities). Here we show that numerous studied aquatic Empididae were characterized by unique habitat preferences and were restricted to the foothills or the lower montane zone with only a few species characterized by wider elevational distribution. *Chelifera pectinicauda, C. flavella, C. subangusta* and *Phyllodromia melanocephala* (Hemerodromiinae), and *Clinocera appendiculata, C. fontinalis, C. wesmaeli, Dolichocephala guttata, D. oblongoguttata, Kowarzia plectrum, Wiedemannia jazdzewskii,* and *W. thienemanni* (Clinocerinae) were clearly associated with the highest altitudes and shaded areas while *W. bistigma, W. lamellata, W. phantasma,* and *W. tricuspidata* (Clinocerinae) were clearly associated with the lower elevated, wider stream valleys overgrown by willow brakes. Species richness and diversity decreased along elevational gradient with the hump-shaped diversity pattern noted for the subfamily Clinocerinae. The altitude, size of river/stream as well as the type of plant community were found as the most important factors in the distribution of the studied aquatic empidid species. The present study is the first one focused on elevational diversity gradient and habitat preferences of Hemerodromiinae and Clinocerinae of central Europe, and one of only a few in the world.

## 1. Introduction

One of the most universal biogeographical patterns of species richness, known as the latitudinal gradient in species diversity, shows the decline of species richness from the tropics towards the Poles. The similarity between climatic factors at high altitudes and high latitudes suggests that the elevational richness gradient reflects the latitudinal richness gradient [1,2]. Many ecological studies reveal two main patterns of relationships between species richness and altitude: a monotonic decline in species richness with increasing elevation, and a hump-shaped relationship, with mid-elevation peaks in species richness [1]. Both models are well documented by studies focused on plants and vertebrates, especially birds and mammals [2,3,4,5,6,7,8,9,10]. Recent studies show that the second pattern seems to be more common [11,12,13,14,15,16,17,18,19,20,21,22,23,24]. On the other hand, relatively little was published on this topic in the case of insects, even if this is the most diverse animal group. Most studies were focused on butterflies, moths, ants, and beetles [18,20,25,26,27,28,29,30,31,32,33,34,35,36]. With respect to the flies, not many studies have been dedicated to the distributional patterns along the elevational gradient, which is usually explained by many difficulties occurring during field sampling [37,38,39,40,41,42]. Moreover, significantly more data are available for terrestrial insects [32,34,35,36] than for the aquatic groups, especially flies [43,44,45,46,47,48]. In addition, most of the studies mentioned above were done in the high mountain habitats located in the tropical regions. Very little is known about Diptera distributional patterns in lower mountain ranges [49], which is sometimes explained by the absence of significant climatic factors differentiating fauna in such mountain massifs including a lack of strict altitudinal zonation. Although more data are needed to confirm such hypothesis, generally it is known that the distribution of many fly species seems to be strictly connected with altitude, with some species occurring only in the higher parts of the mountains, others are known only from the lower elevations while some opportunistic taxa can be found along the entire elevational gradient. 

Although aquatic Empididae is known as a good model group in biodiversity studies [50], current knowledge on elevational patterns of Hemerodromiinae and Clinocerinae flies is surprisingly insufficient and is based only on studies from the Doi Inthanon Mts. located in northern Thailand [51,52,53,54,55,56]. In the case of central Europe, including Poland, knowledge about elevational distribution preferences of aquatic dance flies is rudimentary and is based mainly on faunistic data with no advanced analysis of their ecological preferences [57,58,59,60,61,62,63,64,65,66,67,68,69,70,71,72,73,74,75]. Moreover, there is no detailed study focused on the elevational distribution of flies classified in any of these two subfamilies in lower mountain massifs. 

Taking into account the data about aquatic empidids presented above, we aimed in the present paper to test the following hypotheses: 1/ species richness and distribution of Hemerodromiinae and Clinocerinae species are changing with the elevational gradient, 2/ occurrence of different aquatic dance fly species is correlated with the abiotic and/or biotic environmental parameters like altitude (m), stream mean width (cm), stream mean depth (cm), shading (%), and dominant plant communities at the study sites, 3/ particular Hemerodromiinae and Clinocerinae species prefer similar types of habitat in different study sites. 

## 2. Materials and Methods

### 2.1. Study Area and Field Sampling

The Pieniny Mts. are part of the Outer Western Carpathians and are divided into three smaller regions including Pieniny Właściwe, Little Pieniny (Pieniny Małe), and Pieniny Spiskie [76,77]. The mountain massif is a unique geological unit, composed of a variety of Jurassic and Cretaceous limestone, six kilometres wide and 35 kilometres long, and belongs to the Pieniny Klippen Belt [78,79]. Depending on the definition of the Pieniny Mts., with an average altitude of 630 m, they are classified as a lower or medium mountain massif [80,81]. They can be characterized by the presence of only two altitudinal zones: foothills, which cover small areas and range below ca. 550 m, and lower montane, which cover almost the entire massif. Such low values of height above ground level result in small altitudinal zonation especially in comparison to the higher mountain massifs located close to the Pieniny Mts., including Tatra Mts., Bieszczady Mts., or Babia Góra Mt. The streams in the Pieniny Mts. have various mountain exposure and have typical mountain character even if they are running along a median elevational gradient (410–770 m). In addition, the Dunajec River, which is a large mountain river collecting waters from all streams located in the Polish part of the massif, increases the mosaic of freshwater habitats in this region [82,83,84]. Although the Pieniny Mts. cover a relatively small area (ca. 50 km^2^), they can be characterized by a large mosaic of habitats having different microclimatic conditions that result in a high biodiversity. Based on Witkowski [85], over 7300 animal species and numerous plant species have been noted from this mountain massif, including both boreal and xerothermic taxa as well as glacial relicts or endemics to the Pieniny Mts. [86]. 

**Figure 1:**
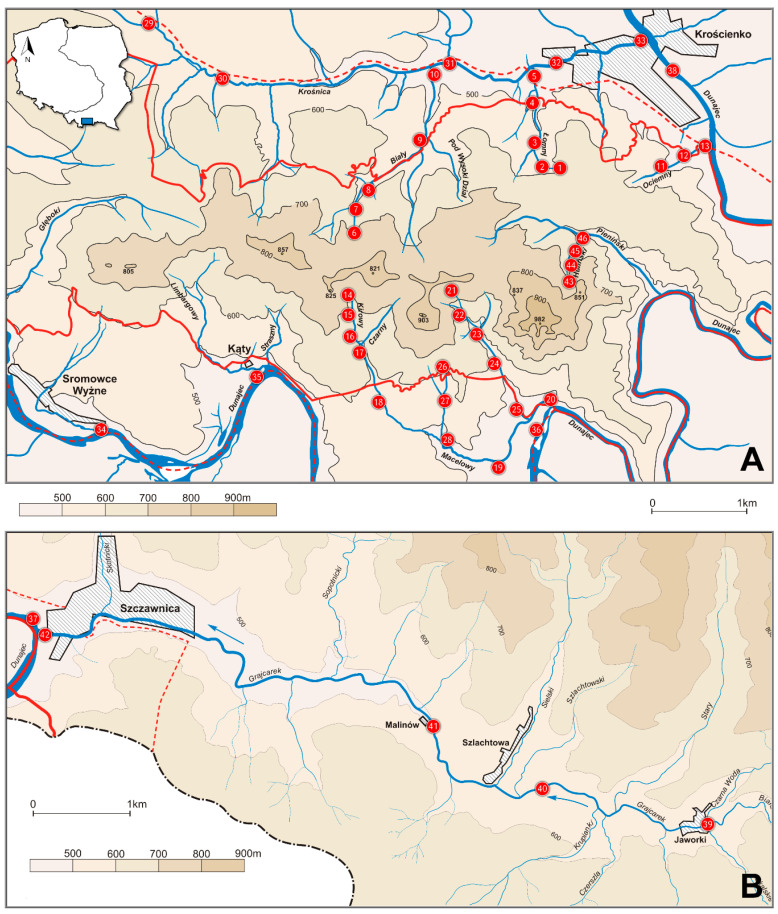
Localities of sampling sites in the Pieniny Mts., Poland: (**A**) Pieniny Właściwe (including Pieniny National Park and its buffer zone), (**B**) Little Pieniny (Małe Pieniny).

The study was carried out in the Polish part of the Pieniny Mts., Southern Poland, including Pieniny National Park, between April 1998 and November 2004. Materials were collected by the senior author (I.S.) on 46 sampling sites located on an elevational gradient between 420 m and 770 m (Table 1, Figure 1).

**Figure 2:**
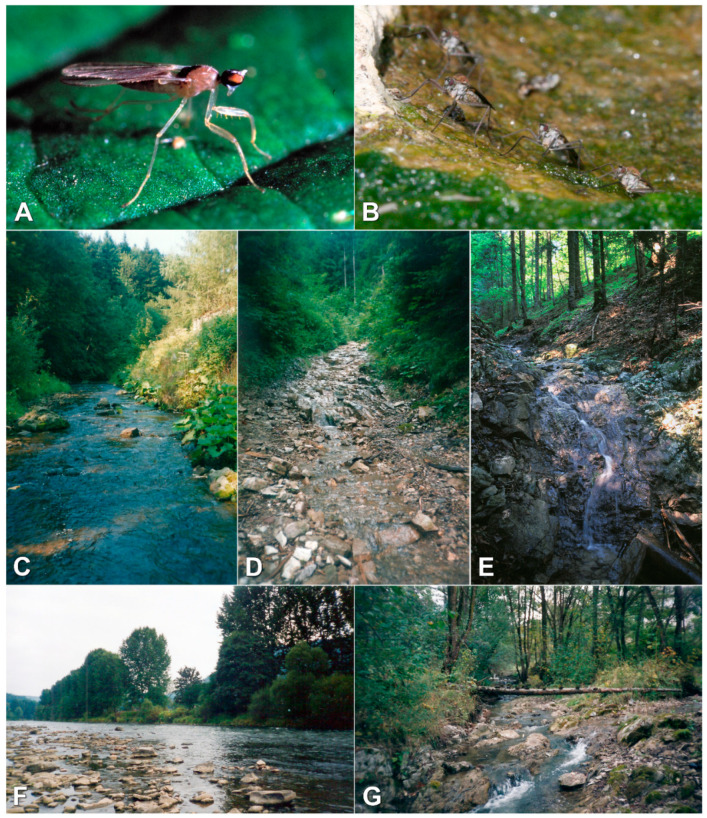
Some empidid species (**A,B**) and their habitats (**C–G**) in the study area. (**A**) Female of *Hemerodromia unilineata* (Hemerodromiinae), (**B**) Group of *Wiedemannia bistigma* (Clinocerinae) on the boulder in the Dunajec River, (**C**) Krośnica stream (sampling site no. 32), (**D**) Sobczański stream (sampling site no. 23), (**E**) Kirowy stream (sampling site no. 15), (**F**) Dunajec River (sampling site no. 38), (**G**) Grajcarek stream (sampling site no. 39) (phot. A—A. Palaczyk, B–G—I. Słowińska).

During the sampling period, each site was visited three times per year: in spring, summer, and autumn. The Hemerodromiinae and Clinocerinae flies were collected along the following streams located in the national park, from headsprings to their outlets: Łonny, Biały, Ociemny, Huliński, Kirowy, Macelowy, Kotłowy, Sobczański, as well as along sections of the Krośnica stream, the Dunajec River (all located in the Pieniny Właściwe Mts.) and the Grajcarek stream (placed in the Little Pieniny Mts.) (Figure 2). The sampling procedure consisted of starting the gathering at the site near the headspring (if applicable), and proceeding downstream over the course of the day. Each stream was sampled over the course of a single day. On each sampling site, both abiotic and biotic environmental parameters were measured including: altitude (m), stream mean width (cm), stream mean depth (cm), shading (%), and dominant plant communities at the study site. The width and depth of the streams and rivers were measured each time material was sampled. In the analysis, only average values were used. Adults of Hemerodromiinae and Clinocerinae were captured using a sweep net or collected with tweezers (directly from stones protruding from the water, moss overgrowing rocks, etc.). At each sampling site, flies were collected for ca. 90 minutes. During the first half of the sampling period, materials were collected from emergent stones. The second 45 minute period was focused on sampling from vegetation along the banks of the stream. Both shaded and sunny areas were checked for Clinocerinae and Hemerodromiinae species presence. To exclude the potential impact of weather on the aquatic empidids activity, samples were not collected during rainfall. Each sampling site was defined as a length of ca. 20 m of the stream measured on both of its sides. The material (in total 42,155 individuals) was preserved in 75% ethanol and later identified in the laboratory by the senior author (I.S.); currently, it is deposited in the Department of Invertebrate Zoology and Hydrobiology of the University of Lodz (Łódź, Poland).

### 2.2. Statistical Methods

The numbers of species and individuals for the two main altitudinal zones – foothills (F) and lower montane (LM) were accounted separately for Hemerodromiinae and Clinocerinae flies. To examine the elevational distribution of both subfamilies, the altitudinal ranges were divided into eight bands, each with an altitudinal value of 50 m: 400–450 m (B1), 451–500 m (B2), 501–550 m (B3), 551–600 m (B4), 601–650 m (B5), 651–700 m (B6), 701–750 m (B7), and >750 m (B8) (Table 2). In order to determine the constancy of occurrence for each species, the frequency was used, which expressed the relation of the number of sites where the species occurred to the number of all the studied sites. Five classes of frequency were distinguished: euconstants > 75.0%, constants 55.1–75.0%, subconstants 35.1–55.0%, accessory species 15.1–35.0%, and accidents <15.0%. The diversity was evaluated using the following indices: species richness (S), the Shannon Index (H’), the Simpson Index (D), and Margalef’s (*D_Mg_*) and Pielou Evenness (J’) indices.

Forty Empididae species (16 Hemerodromiinae and 24 Clinocerinae) collected in 46 samples from the Pieniny Mountains were used in the multivariate analysis. All multivariate analyses were based on square-root transformed semiquantitative biotic data. Additionally, 17 environmental variables were measured (1) abiotic: altitude (m), stream mean width (cm), stream mean depth (cm), shading (%), and (2) biotic-dominant plant communities at the study site on a 0–3 scale: beech mountain forest (B), fir mountain forest (J), eutrophic brook flora (EMG), alder forest (O), burdock brake (LOP), rocky grasslands (MN), pasture (P), meadow (LK), tree plantations (DN), willow brake (ZW), sycamore mountain community altitudinal zones forests (JAW), xerothermic grasslands (MKS), and crop fields (PU).

The SIMPER analysis was conducted using a Bray-Curtis similarity index and 100% cut off for low contributions as a proposal to find patterns of species distribution in the Pieniny Mts. The SIMPER analysis was separately calculated for Hemerodromiinae and Clinocerinae subfamilies, comparing species composition between two sample groups: F (foothills ≤ 550 m) and LM (lower montane zone > 550 m).

To obtain the main environmental factors influencing dipteran communities, an ordination analysis was conducted. The detrended correspondence analysis (DCA) was calculated with detrending by segments and downweighting rare species, to recognize the data distribution (linear or unimodal). As the lengths of the gradient for the first and second DCA axes were respectively 5.523 SD and 5.259 SD units, a canonical correspondence analysis (CCA) was calculated for environment and biota variables comparison. CCA was calculated on inter-species distance with Hill’s scaling anddownweighting rare species. As there was no autocorrelation between environmental variables, they were all included in the analysis. The full model, unrestricted Monte-Carlo permutation test was calculated using automatic selection to indicate the significance of the relation of biota-environmental variables. Because CCA triplot revealed the arch effect, a detrended canonical correspondence analysis (DCCA) was also conducted with detrending by segments and downweighting rare species. Unrestricted Monte-Carlo permutation test was performed to obtain the significance of the first ordination axis under the full model.

The multivariate analysis was calculated using PRIMER 6 and Canoco 4.5 software [87,88].

## 3. Results

### 3.1. Species Composition, Species Richness and Diversity along the Elevational Zones 

In total 42,155 individuals belonging to 40 species of aquatic empidids have been collected from 46 sampling sites from the Pieniny Mts. Among them, eleven species are known as rare including four currently recognized as endemics to this mountain massif [61,63,89]. Aquatic empidids were decidedly dominated by the subfamily Clinocerinae (almost 98% total fauna) which was represented by 24 species grouped in four genera: *Clinocera* Meigen, 1803 (3 species), *Dolichocephala* Macquart, 1823 (3 species), *Kowarzia* Mik, 1881 (1 species), *Wiedemannia* Zetterstedt, 1838 (17 species). The subfamily Hemerodromiinae was represented by 16 species classified in four genera: *Chelifera* Macquart, 1823 (11 species), *Chelipoda* Macquart, 1823 (1 species), *Hemerodromia* Meigen, 1822 (3 species), and *Phyllodromia* Zetterstedt, 1837 (1 species). The most abundant species were *Wiedemannia bistigma* with a total of 22,137 (53.8% of Clinocerinae fauna) and *Chelifera trapezina* with a total of 271 (26.6% of Hemerodromiinae fauna). In total, almost 60% of all aquatic empidids collected in the study were caught in the Dunajec, the major river in the Pieniny Mts., with *Wiedemannia bistigma* as the most abundant species recorded at all sampling sites along this river (19,038 individuals or 46.3% of clinocerines). Nine species were represented by less than 10 individuals, three species were singletons and two species were doubletons. 

**Figure 3:**
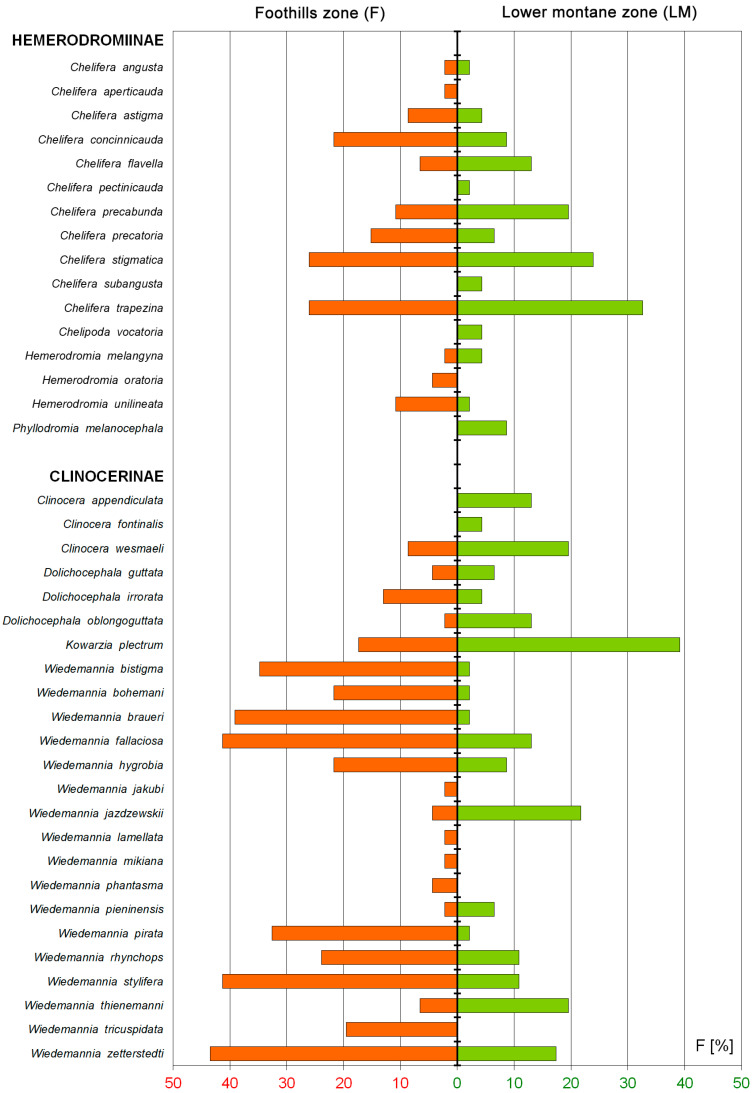
Frequency of Hemerodromiinae and Clinocerinae at the foothills zone and the lower montane zone in the Pieniny Mts.

The distribution of aquatic empidids by the altitudinal zones shows that 12 Hemerodromiinae and 22 Clinocerinae species occur at the foothills zone (F) while at the lower montane zone (LM) these groups are represented by 14 and 19 species respectively (Figure 3, Table 2). For both families, the highest number of individuals was recorded at the foothills (650 of Hemerodromiinae and 38,732 of Clinocerinae). Seven species (17.5% of total fauna) were found exclusively at the foothills including *Chelifera aperticauda*, *Hemerodromia oratoria* (Hemerodromiinae), *Wiedemannia jakubi*, *W. lamellata*, *W. mikiana*, *W. phantasma*, and *W. tricuspidata* (Clinocerinae) while six species (15%) were restricted to the lower montane zone: *Chelifera pectinicauda*, *C. subangusta*, *Chelipoda vocatoria*, *Phyllodromia melanocephala* (Hemerodromiinae), *Clinocera appendiculata,* and *C. fontinalis* (Clinocerinae). Another five aquatic empidids were found as more opportunistic taxa which can occur at both the foothills and the lower montane zone (*Chelifera precabunda*, *C. stigmatica*, *C. trapezina*, *Dolichocephala guttata*, *Wiedemannia pieninensis*) (Table 2).

**Figure 4:**
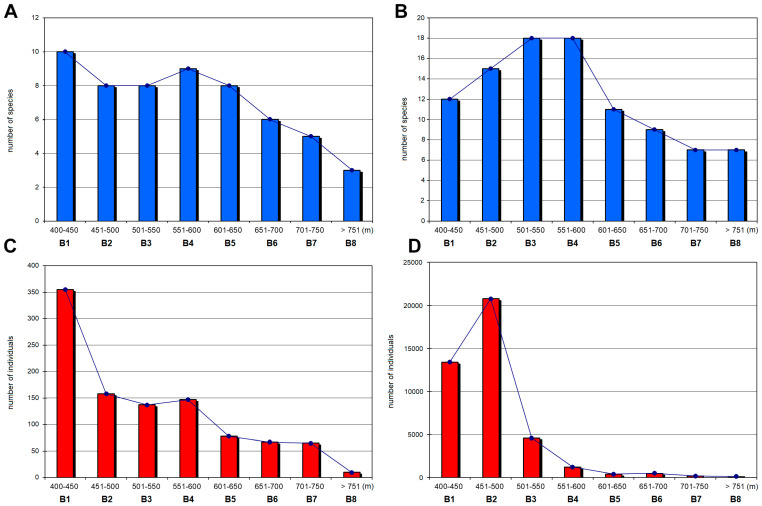
Species richness (**A,B**) and the number of individuals (**C,D**) along the altitudinal bands calculated for the subfamilies Hemerodromiinae (**A**,**C**) and Clinocerinae (**B**,**D**).

Species richness of the Clinocerinae subfamily gradually increased with altitude with a peak at the middle ranges, 501–550 m (B3) and 551–600 m (B4), and then decreased with increasing elevation (Figure 4). Although for these two bands the highest species richness was recorded, it was only in B4 that the highest values of Shannon’s, Simpson’s, Margalef’s and Pielou Evenness indices were noted (Table 3). This was reflected in the large number of species (18) recorded at this altitude interval, which decreased with the further increase in elevation to only seven species above 750 m (B8). On the other hand, at 451–500 m (B2) there were only three fewer species but all of the estimated indices, except Margalef’s, were lower than at 551–600 m (B4). It is worth noting that over 77% of the individuals found on the entire altitudinal range B2 (451–500 m) belong to one species, *Wiedemannia bistigma* (the most abundant clinocerine species found by us in the Pieniny Mts.).

In examining the species richness of the subfamily Hemerodromiinae across each elevational band, a downwards trend with increasing altitude was apparent, although species richness was the highest at the lowest zone (400–450 m; B1). The results of all estimated indices for this band were relatively lower compared with those obtained at higher elevations (Table 3). 

Overall, both subfamilies were more abundant at the two lowest elevational zones (B1—Hemerodromiinae, B2—Clinocerinae). These intervals generated the greatest number of individuals but did not generate a correspondingly greater number of species. This could be due to the location of sites along the Dunajec River, which provide the maximum number of clinocerines. Approximately 24,236 of the individuals collected from this river comprised three species: *Wiedemannia bistigma*, *W. braueri,* and *W. tricuspidata*. There was a significant increase in clinocerine abundance at the band B2 (451–500 m). Increased clinocerine numbers at this interval were mainly due to the presence of *W. bistigma* at each site along Dunajec River. 

The highest number of Hemerodromiinae individuals were recorded at the lowest elevation (B1) with 355 individuals of which more than half was *Chelifera concinnicauda*. In the next elevational band (B2) a dramatic decrease of Hemerodromiinae individuals was noted. Above 750 m (B8), both species richness and the number of individuals clearly decreased (Figure 4). Moreover, the values of calculated indices were relatively low. 

Both subfamilies were not evenly distributed along the altitudinal ranges (Table 2). *Wiedemannia bistigma*, *W. braueri*, *W. tricuspidata*, *W. lamellata,* and *W. phantasma* were the main species contributing to the low-elevation communities (below 550 m). All these species occurred mainly along the Dunajec River and Krośnica stream (both placed at the foot of the Pieniny Mts.), and also in the lower stream sections but only in the outlets to the Dunajec River. The most abundant Clinocerinae species,—*W. bistigma*, *W. bohemani*, *W. braueri*, *W. pirata*, *W. rhynchops*, *W. stylifera* and *W. tricuspidata*—had a similar distribution pattern of decreasing numbers with altitude. Interestingly, all mentioned species were absent above 600 m, whereas *W. tricuspidata* did not exceed 500 m. 

Among the Hemerodromiinae, two species—*Chelifera concinnicauda* and *C. stigmatica—*were the main contributing species to the low-elevation communities. 

For the high elevations, *Clinocera appendiculata*, *C. fontinalis*, *Dolichocephala guttata*, *D. oblongoguttata*, *Kowarzia plectrum*, *Wiedemannia jazdzewskii*, and *W. thienemanni* (Clinocerinae), as well as *Chelifera flavella*, *C. pectinicauda*, *C. subangusta*, *Chelipoda vocatoria*, and *Phyllodromia melanocephala* (Hemerodromiinae) were the main contributing species. However, *Chelifera trapezina* was found to occur in all elevational intervals and it was most abundant in the middle elevation (551–600 m; B4). 

Some species were noted only in one interval, including *Chelifera aperticauda*, *Hemerodromia oratoria*, and *Wiedemannia phantasma* in band 1 (400–450 m), *W. lamellata* in band 2 (451–500 m), *W. jakubi* and *W. mikiana* in band 3 (501–550 m), and *Chelifera pectinicauda* in band 8 (>750 m).

Others, even though occurring throughout almost all elevational ranges, presented a higher abundance in certain altitudes, such as *C. precabunda*, *C. trapezina*, *C. wesmaeli,* and *Wiedemannia thienemanni* at the middle range (551–600 m; B4), *Kowarzia plectrum* at 651–700 m (B6), *Wiedemannia jazdzewskii* at 601–650 m (B5), and *W. zetterstedti* at 451–500 m (B2). 

### 3.2. Biotic and Abiotic Factors vs. Distribution at the Altitudinal Zones 

**Figure 5:**
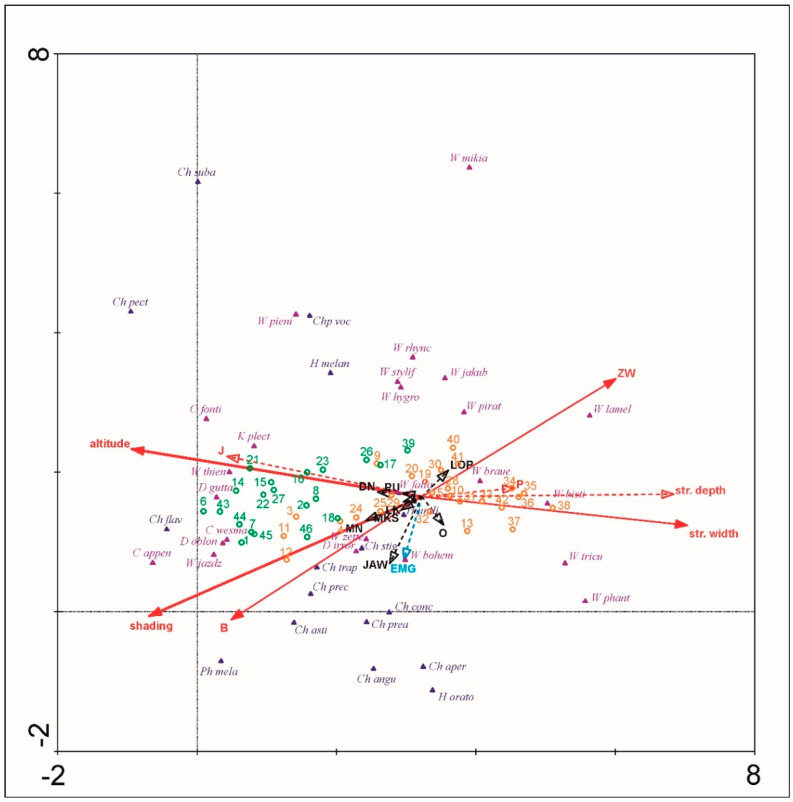
Plot of the detrended canonical correspondence analysis (DCCA) results. Red arrows—environmental variables correlated with 1st DCCA axis, blue arrow – environmental variable correlated with 2nd DCCA axis, black arrows—environmental variables not correlated with first two DCCA axes. Canonical correspondence analysis (CCA) results remarked on the DCCA plot: Thick solid arrows—environmental variables significantly explaining biota distribution with CCA λ1:λ2 > 1, thin solid arrows—environmental variables significantly explaining biota distribution with CCA λ1:λ2 < 1, dashed arrows—not significant variables. Sample number remarks: orange—altitude ≤ 550 m; green—altitude > 550 m. Environmental symbols: str. depth—stream mean depth (cm), str. width—stream mean width (cm), B—beech mountain forest, EMG—eutrophic brook flora, J—fir mountain forest, O—alder forest, LOP—burdock brake, MN—rocky grasslands, P—pasture, LK—meadow, DN—tree plantations, ZW—willow brake, JAW—sycamore mountain forests, MKS—xerothermic grasslands, PU—crop fields. Taxa name abbreviations—Hemerodromiinae (navy blue): *Chelifera angusta* (*Ch angu*), *C. aperticauda* (*Ch aper*), *C. astigma* (*Ch asti*), *C. concinnicauda* (*Ch conc*), *C. flavella* (*Ch flav*), *C. pectinicauda* (*Ch pect*), *C. precabunda* (*Ch prec*), *C. precatoria* (*Ch prea*), *C. stigmatica* (*Ch stig*), *C. subangusta* (*Ch suba*), *C. trapezina* (*Ch trap*), *Chelipoda vocatoria* (*Chp voc*), *Hemerodromia melangyna* (*H melan*), *H. oratoria* (*H orato*), *H. unilineata* (*H unili*), *Phyllodromia melanocephala* (*Ph mela*); Clinocerinae (violet): *Clinocera appendiculata* (*C appen*), *C. fontinalis* (*C fonti*), *C. wesmaeli* (*C wesma*), *Dolichocephala guttata* (*D gutta*), *D. irrorata* (*D irror*), *D. oblongoguttata* (*D oblon*), *Kowarzia plectrum* (*K plect*), *Wiedemannia bistigma* (*W bisti*), *W. bohemani* (*W bohem*), *W. braueri* (*W braue*), *W. fallaciosa* (*W falla*), *W. hygrobia* (*W hygro*), *W. jakubi* (*W jakub*), *W. jazdzewskii* (*W jazdz*), *W. lamellata* (*W lamel*), *W. mikiana* (*W mikia*), *W. phantasma* (*W phant*), *W. pieninensis* (*W pieni*), *W. pirata* (*W pirat*), *W. rhynchops* (*W rhync*), *W. stylifera* (*W stylif*), *W. thienemanni* (*W thien*), *W. tricuspidata* (*W tricu*), *W. zetterstedti* (*W zette*).

The SIMPER analysis indicated that Clinocerinae and Hemerodromiinae communities reveal clear dissimilarity (88.81% and 82.46% respectively) for the foothills (F) and the lower montane (LM) altitudinal zones. The communities of both altitudinal zones have also low specific similarity (Clinocerinae: F = 28.6%, LM = 16.31%; Hemerodromiinae: F = 13.84%, LM = 28.76%). Among the three Clinocerinae taxa that contribute mostly to the foothills (F) communities, only *Wiedemannia zetterstedti* was also a common dominant at the lower montane zone (LM). *W. braueri* and *W. bistigma* were specific for F and *W. jazdzewskii* with *W. thienemanni* remaining specific for the LM. Otherwise, among Hemerodromiinae, the two most contributing taxa for the F and LM—*Chelifera trapezina* and *C. stigmatica*—were the common dominants for both altitudinal zones. Among the three main dominants, only *Chelifera concinnicauda* and *C. flavella* remained specific for the F and LM respectively. Using the environmental variables, the first and second CCA axes explained respectively 22.7% and 11.9% of species data percentage variance as well as 41.7% and 21.9% of species-environment relation percentage variance (Figure 5). Five of seventeen environmental variables significantly explained biota variance: altitude (*p* = 0.002, explaining 16.7% of biota variance, λ1:λ2 = 1.17), stream mean width (*p* = 0.002, explaining 9.3% of biota variance, λ1:λ2 = 0.83), B (*p* = 0.002, explaining 5.25% of biota variance, λ1:λ2 = 0.58), ZW (*p* = 0.002, explaining 4.0% of biota variance, λ1:λ2 = 0.50), and shading (*p* = 0.01, explaining 3.4% of biota variance, λ1:λ2 = 1.02). The first and second DCCA axes explained respectively 22.7% and 3.5% of species data percentage variance as well as 40.7% and 8.1% of species-environment relation percentage variance. The test of significance of the first canonical DCCA axis revealed eigenvalue = 0.736, F-ratio = 8.233, and *p*-value = 0.002. Stream mean width, stream mean depth, P and ZW were positively correlated with DCCA axis 1. Altitude, shading, B, and J were negatively correlated with DCCA axis 1. Only insignificant to CCA Monte-Carlo test, EMG was negatively correlated with DCCA axis 2. Environmental plant community variables O, LOP, MN, LK, DN, JAW, MKS, and PU were not correlated with the first two DCCA axes. Communities typical to the LM zone were distributed only at a higher altitude, shading, and B. Communities from the F zone were distributed generally at a lower altitude and shading and were spread along wider streams with willow vegetation. Only samples from 12, 11, 3, and 4 P sites, were localized at the shaded beech forests (B) representing also higher altitudes (463–520 m). *Chelifera pectinicauda, C. flavella, C. subangusta,* and *Phyllodromia melanocephala* among Hemerodromiinae, and *Clinocera appendiculata, C. fontinalis, C. wesmaeli, Dolichocephala guttata, D. oblongoguttata, Kowarzia plectrum, Wiedemannia jazdzewskii,* and *W. thienemanni* among Clinocerinae are taxa clearly associated with the highest altitudes and shading in the Pieniny Mountains. They are also frequent in beech forests. *W. bistigma, W. lamellata, W. phantasma,* and *W. tricuspidata* from the Clinocerinae are clearly associated with lower elevated, wider stream valleys overgrown by willow brakes that give less shading than LM beech mountain forests. Other taxa revealed more or less intermediate preferences according to the environmental variables measured. *Chelifera subangusta, Hemerodromia oratoria,* and *Wiedemannia mikiana* revealed a strong/stronger correlation with the 2nd DCCA axis than the 1st DCCA axis. Interestingly, Hemerodromiinae revealed lower 2nd DCCA axis values whereas for Clinocerinae, more frequently significant the 2nd CCA axis variance came out. Other, not included factors (maybe some like EMG i.e., soil/water chemistry, trophy, or ground/stream bank microhabitat quality) may determine the distributional pattern of these two subfamilies in the Pieniny Mts.

## 4. Discussion

### 4.1. Species Richness of the Pieniny Mts. vs. Polish and European Mountain Massifs

The subfamilies Hemerodromiinae and Clinocerinae belong to a large family Empididae, distributed worldwide. They are far more common in temperate localities and mountainous areas. In the present study, 40 species classified in these subfamilies were recorded in the Pieniny Mts. which makes up 62.5% of Polish fauna [57,58,63,65,67,68,71,72,74,89]. One species, *Wiedemannia pieninensis*, until recently was known only from the Pieniny Mts., but lately it was recorded in the Tatra Mountains [67]. As a result, currently in the Pieniny Mts., the highest number of aquatic empidids is known compared with other Polish mountain massifs where only 29 species have been noted for the Tatra Mts., 28 for the Gorce Mts., 24 species for both the Bieszczady Mts. and the Babia Mt., and 15 for the Świętokrzyskie Mts. [57,61,62,63,67,70,71,72,90,91]. Moreover, significantly lower numbers of aquatic Empididae have been noted also from some much bigger and higher mountain massifs including the Sierra Nevada Mts. in Spain (24 species) [92] or the Caucasus Mts. (31 species) [93,94,95,96,97].

Our study also shows new altitudinal limits for some of the recorded species. One of them is *Clinocera appendiculata*, which in the Pieniny Mts. was noted at 590–770 m (Table 2; Table 4), which is very surprising because this species is known to occur only in high mountains, e.g., it was found by Vaillant [98] in the French Alps at an altitude up to 2560 m at the foot of the glacier des Grands Couloirs (north of Pralognan). In the Pieniny Mts., most individuals of this species were found on the boulders covered by moss mats below the spring of the Biały stream (750 m) located on the northern slopes of this massif [89]. One more interesting species is *Chelifera stigmatica* which was known only from the Poland headsprings up to 1000 m [57] while in the present study it was absent in the samples collected in the springs and the upper stream sections. In contrast, it was the most abundant at the foothills below 550 m. Generally for numerous taxa like *Chelifera flavella*, *C. precabunda*, *C. precatoria*, *C. trapezina*, *Phyllodromia melanocephala* (Hemerodromiinae) and *Clinocera appendiculata*, *C. wesmaeli*, *Dolichocephala guttata*, *D. irrorata*, and *K. plectrum* (Clinocerinae), the highest elevational values of their distribution in the Pieniny Mts. correspond with the lowest elevational values of their distribution observed in other European mountain massifs. Such results clearly suggest significant adaptability of some species of Hemerodromiinae and Clinocerinae to environmental factor changes including habitats and altitude. 

### 4.2. Elevational Patterns of Hemerodromiinae and Clinocerinae 

The present paper shows the first comprehensive analysis of the elevational distribution of aquatic Empididae in central Europe and it is also one of the few studies focused on this group of Diptera in the world [51,52,53,54,55,56].

Our study on the distribution of Hemerodromiinae and Clinocerinae along an elevational gradient indicate the richness and abundance decreases with altitude which is rather typical in the elevational distribution of different insect groups including e.g., beetles [20,29,31,32,34,36], butterflies [25,33,35], moths [18,28], or ants [26,27,30]. Comparing the abundance of both subfamilies across eight elevational bands, a clear downwards trend with increasing altitude was apparent. A pronounced reduction in the number of Hemerodromiinae individuals was found from 400–450 m up, and only 10 individuals were recorded above 750 m. A dramatic decline in the number of individuals with increasing altitude was also revealed for Clinocerinae. For both subfamilies, a distinct species-poor community was recorded at the highest elevation (>750 m), which also corresponds with numerous other studies focused on mountain insect fauna [18,20,26,27,28,29,30,31,32,33,34,35,36] and usually can be explained by the most extreme environmental conditions observed on the tops of mountain massifs.

Mid-elevation and low elevation peaks in species richness have been observed in different insect groups [11,17,99]. Our study data for Hemerodromiinae strongly supported a low elevation peak (400–450 m) in species richness and monotonic decline in species richness with an increase of altitude. In contrast, our findings do not confirm the results of studies on this subfamily at Doi Inthanon, Thailand where the total number of taxa and individuals increased with altitude, declining just above a maximum of 2200 m [52]. On the other hand, we found that in the Pieniny Mts., the species richness of the subfamily Clinocerinae shows a slightly hump-shaped pattern with a peak of 18 species and the highest values of all diversity indices at a mid-elevational range (551–600 m). Interestingly, consideration of the elevational distribution of singletons and doubletons indicated that rare species contribute to species richness at both low and higher elevation in the Pieniny Mts. Our results for clinocerines corresponds with data received for many other insect groups studied in different mountain areas of the world [13,18,19,20,21,23,24], even if a great percentage of these studies were made in much higher mountain massifs than the Pieniny Mts. In contrast to our study, a different trend was found for some other aquatic and terrestrial invertebrate groups studied in Polish mountain massifs including Mount Babia, Tatra Mts., and Bieszczady Mts. as both species richness and number of individuals decreased with increasing altitude [100,101,102,103,104,105]. In addition, in Doi Inthanon, northern Thailand where the subfamily Empidinae [56] was studied, which is closely related to both empidid subfamilies in our study, a linear increase in abundance and species richness was observed along the elevational gradient. Moreover, Plant et al. [106] noted similar results during a study upon the superfamily Empidoidea in the same area in Asia, as they found that genus richness of Empididae, Hybotidae, and Brachystomatidae increased with elevation, while only for the Dolichopodidae, genus richness was the highest at mid-elevations.

It is assumed that habitat complexity contributes to an increase in species diversity and abundance in many groups of organisms, including aquatic insects [107,108,109,110]. Our study shows that species richness and species composition of Hemerodromiinae and Clinocerinae vary along the altitudinal gradient with a peak at the foothills (<550 m) and decline at the lower montane zone (>550 m). Data from the foothills shows that the highest number of clinocerines was collected along the Dunajec River valley, which is a major river in the Pieniny Mts., although the species richness was lower than in sites located along smaller streams. Most probably this can be explained by a higher mosaic of microhabitats observed in higher elevations which are more attractive for many aquatic empidid flies. Species with high abundances at lowest elevations, e.g., *Wiedemannia bistigma*, *W. braueri*, *W. pirata*, *W. stylifera,* and *W. tricuspidata*, prefer large streams and medium-sized rivers flowing at altitudes between 400 and 550–600 m. The number of these flies generally decreases with elevation. Moreover, changes in the aquatic empidid fauna along the elevational gradient in the Pieniny Mts. could be due to altitudinal species replacement e.g., *Wiedemannia bistigma*, *W. braueri*, and *W. tricuspidata* were restricted mainly to the Dunajec River and Grajcarek stream (both located below 550 m) and were replaced by species that prefer the stream valleys (e.g., *Clinocera wesmaeli*, *Kowarzia plectrum*, *Wiedemannia bohemani*, *W. jazdzewskii*, *W. thienemanni*, and *W. zetterstedti*). Similar results, where some taxa are restricted to the lower parts of mountain massif and others occur only in its higher regions are commonly noted in different insect groups. Usually, it is explained by different habitat preferences of particular species and is known as one of the standard distributional models [11].

Although different factors affect the occurrence and distribution of insects in a different way, usually, many of them play a role at the same time. In our study, 17 environmental factors were tested, of which only five (also as different combinations) were found to significantly regulate Clinocerinae and Hemerodromiinae distribution at the foothills and the lower montane zone in the Pieniny Mts. Although for some species, lower elevation, less shaded sites and wider streams (or river) with willow vegetation (e.g., *Wiedemannia bistigma*, *W. lamellata*, *W. phantasma*, *W. tricuspidata*) or higher elevation and heavily shaded sites associated with beech mountain forest (e.g., *Chelifera pectinicauda*, *C. subangusta, Clinocera appendiculata*, *C. fontinalis*, *Kowarzia plectrum*, *Wiedemannia jazdzewskii*) were found as the most important factors describing their habitat preferences. For some other aquatic empidids, additional environmental parameters should be studied to clarify their distributional patterns. Such a list can include e.g., stream geomorphology, substratum, water current, pH of water, relative humidity as well as competition between species, as all these factors can play important role in the occurrence and distribution of aquatic immature stages of Clinocerinae and Hemerodromiinae and, as a consequence, also the adult aquatic empidids. Moreover, as we present the first results about habitat preferences for both subfamilies in Europe, future studies focused on aquatic Empididae in other mountain massifs are recommended to verify ecological preferences of particular species not only along elevational but also along a geographical gradient. 

## 5. Conclusions

The results of this study showed that despite the limited altitudinal range of the Pieniny Mts., there are significant changes in relation to aquatic Empididae abundance and species richness along the elevational gradient, which clearly suggests that even in relatively small mountains, significant changes in insect fauna can be correlated with altitude. Obviously, in many aquatic empidids occurring in the Pieniny Mts., the elevational species ranges are much narrower than compared to the Alps, the Pyrenees, or even the Tatra Mts., but it arises from the relatively low altitude of this Polish mountain massif. Moreover, even in such relatively low mountains, the hump-shaped distributional patterns can be characteristic for at least some insect groups. 

## Figures and Tables

**Table 1:** Sampling sites in the Pieniny Mountains, southern Poland.

No.	Sampling Site	Altitude (m)	GPS Location
1	Łonny stream, headspring	620	49°25′46″ N 20°24′56″ E
2	Łonny stream	560	49°25′51″ N 20°24′46″ E
3	Łonny stream	520	49°25′53″ N 20°24′44″ E
4	Łonny stream	463	49°26′11″ N 20°24′45″ E
5	Łonny stream (outlet to the Krośnica stream)	440	49°26′22″ N 20°24′43″ E
6	Biały stream, headspring	750	49°25′12″ N 20°23′09″ E
7	Biały stream	640	49°25′31″ N 20°23′09″ E
8	Biały stream	560	49°25′38″ N 20°23′14″ E
9	Biały stream	515	49°25′52″ N 20°23′43″ E
10	Biały stream (outlet to the Krośnica stream)	462	49°26′18″ N 20°23′45″ E
11	Ociemny stream, headspring	510	49°25′48″ N 20°25′55″ E
12	Ociemny stream	480	49°25′49″ N 20°25′59″ E
13	Ociemny stream (outlet to the Dunajec River)	445	49°25′54″ N 20°26′19″ E
14	Kirowy stream, headspring	770	49°25′02″ N 20°23′10″ E
15	Kirowy stream	680	49°24′51″ N 20°23′09″ E
16	Kirowy stream	610	49°24′45″ N 20°23′10″ E
17	Macelowy stream	587	49°24′38″ N 20°23′15″ E
18	Macelowy stream	560	49°24′35″ N 20°23′17″ E
19	Macelowy stream, Sromowce Niżne village	460	49°24′04″ N 20°24′35″ E
20	Macelowy stream (outlet to the Dunajec River)	450	49°24′22″ N 20°25′01″ E
21	Sobczański stream, headspring	750	49°25′01″ N 20°24′08″ E
22	Sobczański stream	655	49°24′55″ N 20°24′09″ E
23	Sobczański stream	590	49°24′50″ N 20°24′16″ E
24	Sobczański stream	530	49°24′38″ N 20°24′31″ E
25	Sobczański stream, (outlet to the Macelowy stream), Sromowce Niżne village	455	49°24′19″ N 20°24′45″ E
26	Kotłowy stream, headspring	610	49°24′39″ N 20°24′02″ E
27	Kotłowy stream	570	49°24′31″ N 20°24′01″ E
28	Kotłowy stream (outlet to the Macelowy stream)	490	49°24′10″ N 20°23′59″ E
29	Krośnica stream, Krośnica village	550	49°26′41″ N 20°20′43″ E
30	Krośnica stream, near Hałuszowa village	520	49°26′13″ N 20°21′47″ E
31	Krośnica stream, Tylka village	462	49°26′20″ N 20°23′45″ E
32	Krośnica stream, Krościenko village	440	49°26′23″ N 20°24′49″ E
33	Krośnica stream (outlet to the Dunajec River), Krościenko village	415	49°26′33″ N 20°25′39″ E
34	Dunajec River, Sromowce Wyżne village	475	49°24′03″ N 20°21′12″ E
35	Dunajec River, Kąty village	465	49°24′25″ N 20°22′12″ E
36	Dunajec River, Sromowce Niżne village	455	49°24′22″ N 20°25′01″ E
37	Dunajec River, Szczawnica	430	49°26′26″ N 20°27′34″ E
38	Dunajec River, Krościenko village	420	49°26′27″ N 20°25′50″ E
39	Małe Pieniny, Grajcarek stream, Jaworki village	580	49°24′26″ N 20°33′14″ E
40	Małe Pieniny, Grajcarek stream, Szlachtowa village	540	49°24′37″ N 20°31′48″ E
41	Małe Pieniny, Grajcarek stream	490	49°25′03″ N 20°30′47″ E
42	Małe Pieniny, Grajcarek stream (outlet to the Dunajec River), Szczawnica	430	49°25′27″ N 20°27′35″ E
43	Huliński stream	745	49°24′56″ N 20°25′01″ E
44	Huliński stream	680	49°25′VI″ N 20°25′05″ E
45	Huliński stream	630	49°25′15″ N 20°25′04″ E
46	Huliński stream (outlet to the Pieniński stream)	587	49°25′25″ N 20°25′13″ E

**Table 2:** Elevational distribution of Hemerodromiinae and Clinocerinae along the altitudinal ranges at the foothills and the lower montane zones in the Pieniny Mts., Poland.

Altitude (m)	Foothills Zone (F)				Lower Montane Zone (LM)					
	400–450 (B1)	451–500 (B2)	501–550 (B3)	Total	551–600 (B4)	601–650 (B5)	651–700 (B6)	701–750 (B7)	>751 (B8)	Total
**Number of Hemerodromiinae species**	10	8	8	12	9	8	6	5	3	14
**Number of Hemerodromiinae individuals**	355	158	137	650	147	78	67	65	10	367
*Chelifera angusta* Collin, 1927	3			3	1					1
*Chelifera aperticauda* Collin, 1927	1			1						
*Chelifera astigma* Collin, 1927	1	11	1	13				3		3
*Chelifera concinnicauda* Collin, 1927	184	10	11	205	5	1				6
*Chelifera flavella* (Zetterstedt, 1838)		1	11	12		10		53	8	71
*Chelifera pectinicauda* Collin, 1927									1	1
*Chelifera precabunda* Collin, 1961	15	4	6	25	18	5	4	5		32
*Chelifera precatoria* (Fallén, 1816)	7	6	13	26	4		2			6
*Chelifera stigmatica* (Schiner, 1862)	59	59	48	166	41	4	3			48
*Chelifera subangusta* Collin, 1961							1	1		2
*Chelifera trapezina* (Zetterstedt, 1838)	24	47	46	117	64	30	56	3	1	154
*Chelipoda vocatoria* (Fallén, 1816)						1	1			2
*Hemerodromia melangyna* Collin, 1927			1	1	1	1				2
*Hemerodromia oratoria* (Fallén, 1816)	21			21						
*Hemerodromia unilineata* Zetterstedt, 1842	40	20		60	1					1
*Phyllodromia melanocephala* (Fabricius, 1794)					12	26				38
**Number of Clinocerinae species**	12	15	18	22	18	11	9	7	7	19
**Number of Clinocerinae individuals**	13,387	20,751	4594	38,732	1224	394	497	185	106	2406
*Clinocera appendiculata* (Zetterstedt, 1838)					1	23	5	30	1	60
*Clinocera fontinalis* (Haliday, 1833)					1				1	2
*Clinocera wesmaeli* (Macquart, 1835)		2	17	19	23	1	5	2	8	39
*Dolichocephala guttata* (Haliday, 1833)		2		2		1	2	3		6
*Dolichocephala irrorata* (Fallén, 1816)	4		12	16	2	1				3
*Dolichocephala oblongoguttata* (Dale, 1878)			1	1	1	3	1	1		6
*Kowarzia plectrum* (Mik, 1880)		67	267	334	256	173	439	137	84	1089
*Wiedemannia bistigma* (Curtis, 1834)	5932	16,146	53	22,131	6					6
*Wiedemannia bohemani* (Zetterstedt, 1838)	28	99	28	155	2					2
*Wiedemannia braueri* (Mik, 1880)	3657	2542	1231	7430	57					57
*Wiedemannia fallaciosa* (Loew, 1873)	404	583	1449	2436	85	13				98
*Wiedemannia hygrobia* (Loew, 1858)	10	12	105	127	37	2				39
*Wiedemannia jakubi* Krysiak, 2005			1	1						
*Wiedemannia jazdzewskii* Niesiolowski, 1987			21	21	21	159	34	2	4	220
*Wiedemannia lamellata* (Loew, 1869)		35		35						
*Wiedemannia mikiana* (Bezzi, 1899)			5	5						
*Wiedemannia phantasma* (Mik, 1880)	5			5						
*Wiedemannia pieninensis* Krysiak et Niesiolowski, 2004			4	4	1		4		1	6
*Wiedemannia pirata* (Mik, 1880)	90	40	347	477	31					31
*Wiedemannia rhynchops* (Nowicki, 1868)	4	53	104	161	86					86
*Wiedemannia stylifera* Mik, 1889	57	119	691	867	298					298
*Wiedemannia thienemanni* Wagner, 1982		9	9	18	32	3	6	10	7	58
*Wiedemannia tricuspidata* (Bezzi, 1905)	3084	696		3780						
*Wiedemannia zetterstedti* (Fallén, 1816)	112	346	249	707	284	15	1			300

**Table 3:** Species richness and diversity indices calculated for eight altitudinal zones.

Altitude (m)	Foothills Zone (F)			Lower Montane Zone (LM)				
	400–450 (B1)	451–500 (B2)	501–550 (B3)	551–600 (B4)	601–650 (B5)	651–700 (B6)	701–750 (B7)	>750 (B8)
**Hemerodromiinae**								
Species richness	10	8	8	9	8	6	5	3
Shannon-Wiener Index (*H’*)	1.52	1.6	1.57	1.495	1.49	0.69	0.71	0.64
Simpson’s Index (*D*)	0.68	0.75	0.74	0.71	0.72	0.29	0.33	0.34
Margalef Index (*D_Mg_*)	1.53	1.38	1.42	1.6	1.61	1.19	0.96	0.87
Pielou Evenness Index (*J’*)	0.66	0.77	0.76	0.68	0.72	0.38	0.44	0.58
**Clinocerinae**								
Species richness	12	15	18	18	11	9	7	7
Shannon-Wiener Index (*H’*)	1.28	0.86	1.87	2.03	1.28	0.53	0.87	0.81
Simpson’s Index (*D*)	0.68	0.38	0.79	0.83	0.64	0.22	0.42	0.36
Margalef Index (*D_Mg_*)	1.16	1.41	2.02	2.39	1.67	1.29	1.15	1.29
Pielou Evenness Index (*J’*)	0.52	0.32	0.65	0.7	0.53	0.24	0.45	0.42

**Table 4:** Altitudinal limits for selected Hemerodromiinae and Clinocerinae species in the European massifs (data from Pieniny Mts. compared with data by Vaillant [98,111,112,113], Niesiołowski [57] and Palaczyk et al. [71]).

	Alps	Pyrenees	Massif Central	Tatra Mts	Pieniny Mts.
HEMERODROMIINAE					
*Chelifera astigma*	350–1500			970–1100	445–750
*Chelifera flavella*	700–1700			970–1500	480–770
*Chelifera pectinicauda*	730		800		770
*Chelifera precabunda*	1500–1700			910–1520	445–750
*Chelifera precatoria*	700–1100	802–1850	1200	930–1000	440–680
*Chelifera stigmatica*	250–1000		ca. 1460	960–1050	440–680
*Chelifera trapezina*	700–1400	1150–1300		900–1500	440–770
*Hemerodromia unilineata*	400–1000		320		440–560
*Hemerodromia oratoria*			720		440–445
*Phyllodromia melanocephala*	850–1000			990–1150	560–640
CLINOCERINAE					
*Clinocera appendiculata*	800–2560	1000–2100		900–2040	590–770
*Clinocera wesmaeli*	ca. 1000	1000–1300			463–770
*Dolichocephala guttata*	2300	650–1500		1100	463–750
*Dolichocephala irrorata*		650		900–1620	440–630
*Kowarzia plectrum*	1900			980–1150	445–770
*Wiedemannia bistigma*	215–724	115–350	200–470		415–580
*Wiedemannia bohemani*	215–1000	1600	700–875	920	415–560
*Wiedemannia fallaciosa*	80–1130	1050			415–610
*Wiedemannia hygrobia*	210–1850	1350–2150		900–1650	415–640
*Wiedemannia lamellata*			386		465
*Wiedemannia phantasma*	230–877				420–445
*Wiedemannia zetterstedti*	ca. 1000		217–1123		415–680
